# mRNA Delivery Systems Based on Protein Nanocages: How Far Can We Go?

**DOI:** 10.34133/bdr.0032

**Published:** 2024-05-07

**Authors:** Xinying Wang, Ruimin Gao, Xuan Wang, Juan Zhou, Xian-En Zhang, Feng Li

**Affiliations:** ^1^ State Key Laboratory of Virology, Wuhan Institute of Virology, Center for Biosafety Mega-Science, Chinese Academy of Sciences, Wuhan 430071, China.; ^2^ University of Chinese Academy of Sciences, Beijing 100049, China.; ^3^ Guangzhou Women and Children's Medical Center, Guangzhou Medical University, Guangzhou 510120, China.; ^4^ College of Life Sciences and Health, Wuhan University of Science and Technology, Wuhan 430065, China.; ^5^ Faculty of Synthetic Biology, Shenzhen Institute of Advanced Technology, Shenzhen 518055, China.

## Abstract

Messenger RNA (mRNA) therapeutics hold great potential in the prevention and treatment of many diseases owing to several unique advantages. Delivery of mRNA into target cells is a critical step in mRNA therapy. Efficient and safe delivery systems remain an urgent need. Here, we provide an overview of the current applications of protein nanocages (PNCs), which include different types of PNCs, such as viral capsids, nonviral PNCs, and artificial PNCs, in mRNA delivery. PNCs have the features of uniform size, controllable assembly, modifiable inner and outer surfaces, good biocompatibility, and biodegradability, making them ideal candidates for mRNA delivery. In this review, the properties, loading strategies, and delivery outcomes of each tested PNC are introduced. The challenges faced by PNC-based mRNA carriers are discussed. We also share our perspectives on possible strategies to address these challenges, emphasizing the opportunities brought by emerging technologies and disciplinary convergence.

## Introduction

Messenger RNA (mRNA) serves as an intermediate material in the delivery of genetic information from the nucleus to the cytoplasm for the expression of functional proteins [1]. In recent years, mRNA has emerged as a promising class of drugs for treating various diseases [2]. The use of mRNA in disease treatment offers several benefits. First, unlike DNA, mRNA does not integrate into the host genome, thus reducing the risk of gene integration. Second, mRNA can be delivered in a relatively controlled manner, enabling greater control over transfection efficiency and protein expression. Third, mRNA does not need to enter the nucleus and can be translated in the cytoplasm. Fourth, mRNA is particularly suitable for transient protein expression, such as in gene editing, to minimize off-target effects [3]. On the other hand, there are several challenges in the application of mRNA therapeutics. An outstanding one is the delivery because direct injection of naked mRNA into humans has limited efficacy due to its susceptibility to degradation by extracellular ribonucleases and inefficient internalization [4]. Different delivery systems have been developed to deal with the problem. These include lipid nanoparticles, polymeric nanoparticles, peptides, protamine, polysaccharide particles, and cationic lipids [4,5]. At the same time, there are excellent mRNA delivery systems in nature, such as viruses, after billions of years of evolution. In recent years, there has been a growing interest in using biological nanomaterials for mRNA delivery [6]. For example, viruses have inspired the development of mRNA delivery systems via repurposing viral capsids [7] or designing capsid-like artificial protein nanocages (PNCs) [8].

PNCs are protein nanostructures self-assembled from multiple copies of one or a few kinds of proteins. They have several advantages in the development of mRNA delivery systems. First, their surface area and volume can be optimized to achieve desired drug delivery goals such as specific targeting, high capacity for cargo loading, and efficient cellular uptake [9]. Second, PNCs can protect the cargo from premature degradation and interactions with biological environments, thereby facilitating specific tissue delivery [9]. Third, the typical size range of 20 to 200 nm allows them to stimulate the host's innate and adaptive immune responses [10]. Fourth, protein-based nanocages can be biodegraded in vivo, thus reducing the risk of tissue persistence compared to synthetic materials. Last but not least, PNCs are usually biosynthesized. Thus, it is possible to fabricate mRNA-loaded PNCs in engineered cells in a one-pot manner. Therefore, PNCs hold particular potential in the development of new mRNA delivery systems.

**Fig. 1.**
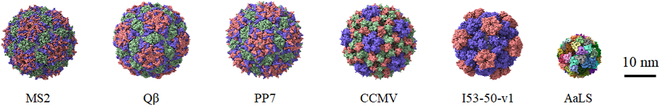
Structure models of PNCs used for mRNA delivery. The protein database IDs of MS2, Qβ, PP7, cowpea chlorotic mottle virus (CCMV), I53-50-v1, and Aquifex aeolicus lumazine synthase (AaLS) are 2BNY, 5VLY, 1DWN, 1ZA7, 7SGE, and 5MPP, respectively.

This review focuses on the current status of mRNA delivery using PNCs, including viral capsids derived from bacteriophages and plant viruses, nonviral natural PNCs, and artificially designed PNCs (Fig. 1). The characteristics, mRNA loading strategies, and delivery outcomes of each PNC are summarized (Table). In addition, the challenges in mRNA delivery by PNCs are discussed.

**Table.** Examples of mRNA delivery using PNCs

PNCs	Origin	Subunits	Diameter (nm)	Cargo mRNA	Packaging strategy	Delivery outcome
MS2	Phage	180	27	mRNA of a prostate cancer antigen	Appending packaging signals on mRNA	Translation in mammalian cells and in vivo [19]
Qβ	Phage	180	28	EGFP mRNA	Similar to above	Translation in mammalian cells and *E. coli* [22]
PP7	Phage	180	26	GFP mRNA	Similar to above	Translation in RM-1 cells [26]
CCMV	Plant virus	180	28	EGFP mRNA	Electrostatic interaction between CP and mRNA	Translation in HEK293T, Hela, and HK2 cells [7]
I53-50-v1	De novo design	120	28	CP mRNA	Directed evolution	Not tested [8]
AaLS	Bacterial enzyme	60	16	CP mRNA	Appending packaging signals on mRNA and directed evolution	Not tested [30]

## Bacteriophage-Based VLPs

Bacteriophages are viruses that infect bacteria. They have a wide range of potential applications in both clinical and nonclinical fields. Compared to eukaryotic viruses, phages are more stable and less prone to genetic changes, making them safer for treating and preventing infections in human and animal populations. Additionally, phage production and purification methods are simple. Based on these properties, phage-based virus-like particles (VLPs) have been developed as nanocarriers [11,12], which are ideal for transferring mRNA into recipient cells for expression [13].

### MS2 VLPs

MS2 phage is a virus with an icosahedral capsid composed of 180 protein subunits. Its single-stranded RNA (ssRNA) genome is 3,569-nucleotide (nt) long [14] and encodes 4 proteins, that is, the coat protein (CP), the mature protein (A-protein), the replicase protein, and the lysis protein [15,16]. The MS2 CP can self-assemble into VLPs in vitro under appropriate conditions. Purified MS2 VLPs can be disassembled by acid treatment and reconstituted by removing acid and adding stem-loop RNA [17]. MS2 VLPs can encapsulate a specific mRNA by interacting with a packaging signal (pac site) located on the mRNA. Appending the MS2 packaging signal to the target mRNA can lead to successful packaging and delivery.

**Fig. 2.**
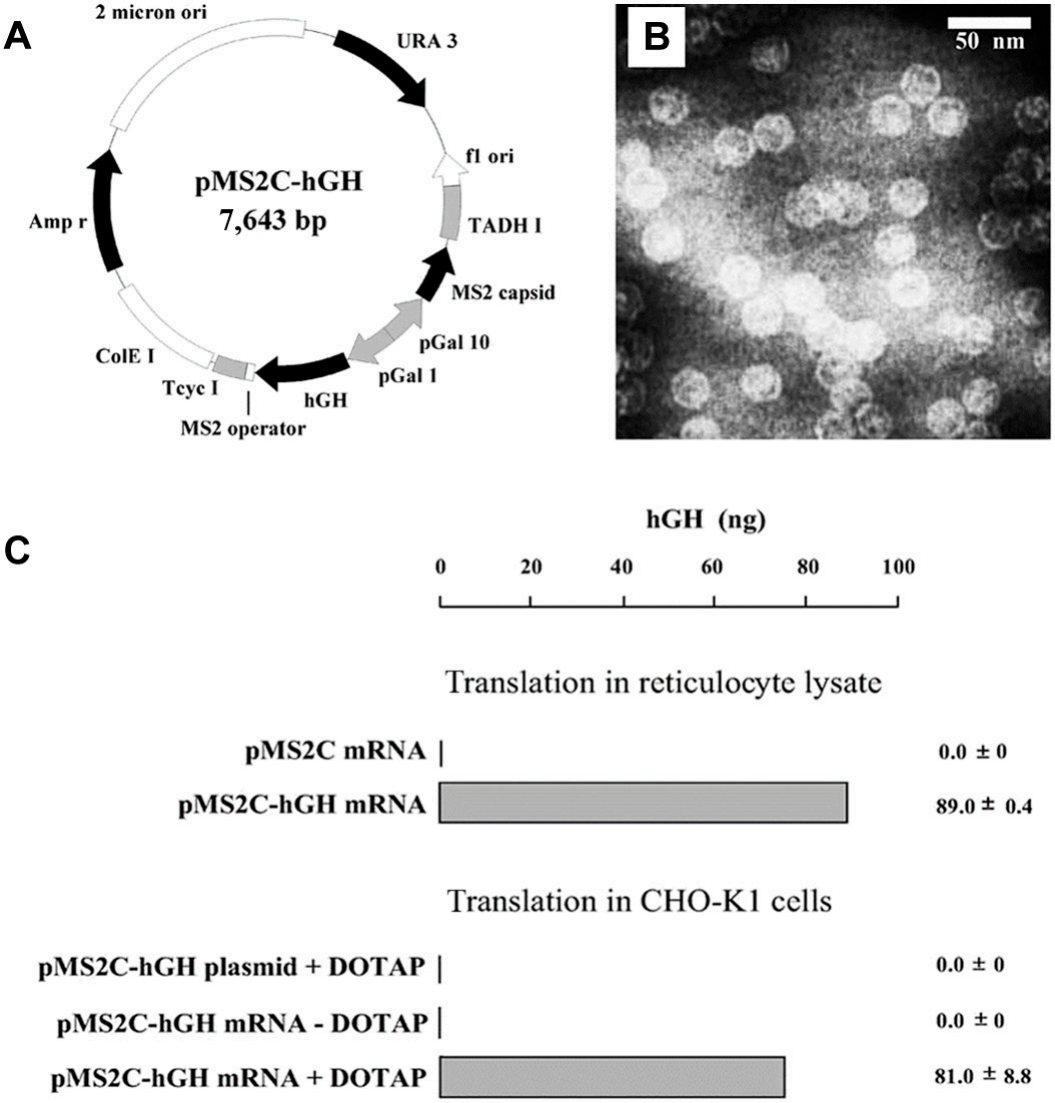
Encapsulation of functional mRNA into MS2 VLPs in yeast. (A) The expression vector of MS2 CP and the cargo hGH mRNA. (B) Transmission electron microscopy image of mRNA-loaded MS2 VLPs. (C) Translation assay of the mRNA purified from MS2 VLPs. Adapted with permission from reference [18].

Legendre and Fastrez [18] expressed MS2 CPs in *Saccharomyces cerevisiae*, which packaged heterologous human growth hormone (hGH) mRNAs with the MS2 packaging signal to form recombinant VLPs. The hGH mRNA was extracted from the VLPs, and then its functionality was verified by in vitro translation and expression assay in cell cultures (Fig. 2). This study opens up the possibility of using MS2 VLPs for mRNA package and delivery. As a step forward with the MS2 nanocarrier, Li et al. [19] developed an MS2 VLP-based mRNA vaccine for the treatment of prostate cancer. Prostatic acid phosphatase (PAP) is known to induce specific cellular immune responses in prostate cancer patients. In this study, a pMS2-mPAP-GM-CSF vector (expressing MS2 CP, mPAP, and GM-CSF simultaneously) was constructed and transformed into the YPH499 yeast strain. Recombinant mRNA-containing MS2 VLPs were synthesized and assembled in the yeast strain through the interaction of MS2 CPs with packaging signal on cargo mRNA. Subsequent characterization showed that the MS2 VLPs could efficiently package target mRNA and can be translated in mammalian cells. The MS2 VLP-based mRNA could induce an effective cellular immune response and delay tumor growth in mouse models.

### Qβ VLPs

Qβ bacteriophage has an icosahedral capsid composed of 180 CP subunits surrounding an RNA genome of approximately 4,217 nt [20]. The self-assembly of Qβ VLPs requires the interaction of stem-loop hairpin secondary structures in the RNA genome with CPs [17]. VLPs can be disassembled into dimers under acidic conditions, and exogenous RNA molecules can be packaged into VLPs by increasing the pH of the solution and adding RNA with a stem-loop structure [21].

Gorzelnik et al. [22] replaced the CP coding sequence in the Qβ genome with the enhanced green fluorescent protein (EGFP) gene and provided the CP in trans with another plasmid. The resultant recombinant VLPs contained the modified genome and could transfect *Escherichia coli* (*E. coli*) to enable EGFP expression. It was unexpected that incubation of the VLPs with Hela cells also led to EGFP expression, as shown by fluorescence microscopy, because the EGFP mRNA was transcribed in a prokaryotic system and did not have the necessary elements for translation in eukaryotic cells. The finding is interesting and requires further investigation to exploit the potential of Qβ VLPs for mRNA delivery in mammalian cells.

### PP7 VLPs

Bacteriophage PP7 is an ssRNA virus with a genome of 3,588 nt [23]. Similar to MS2, the VLPs derived from PP7 can also be expressed in *E. coli.* PP7 CPs can self-assemble into VLPs in the absence of genomic RNA. The β-hairpin structure at the N-terminus of CPs on the surface of PP7 VLPs can tolerate fusion with exogenous peptides to meet varied targeting needs [24,25].

PP7 VLPs can encapsulate exogenous mRNA with packaging signals. By using a dual-expression vector, Sun et al. recombinantly expressed an engineered single-chain CP dimer of PP7 carrying a low-molecular-weight protamine peptide in *E. coli*, which was subjected to the encapsulation of green fluorescent protein (GFP) mRNA containing packaging signals. The resultant VLPs showed high yield, good thermal stability, and easy purification. The successful expression of GFP was observed by confocal laser scanning microscopy, after incubating the VLPs with mouse prostate cancer cells (RM-1 cells) for 24 h. This study demonstrated that PP7 VLPs carrying low-molecular-weight protamine peptides can penetrate cell membranes and deliver packaged mRNA into mammalian cells for translation into mature proteins. Therefore, recombinant PP7 VLPs offer an alternative system for the delivery of mRNA drugs [26].

## Plant Virus-Based VLPs

In addition to bacteriophages, VLPs derived from plant viruses have also been explored for mRNA package and delivery. Compared to synthetic nanoparticles, plant virus delivery systems exhibit higher stability in biological media and are less likely to interact with serum proteins. Their immunogenicity can be reduced through the use of stealth or camouflage coatings. VLPs of many plant viruses have been investigated for various biomedical purposes [27,28]. However, only the VLPs of cowpea chlorotic mottle virus (CCMV) have been examined for mRNA delivery.

CCMV is a widely studied plant virus in the bromoviridae family. The capsid of CCMV is composed of 180 protein subunits with an outer and inner diameter of 28 and 18 nm, respectively [9]. The VLPs of CCMV can be recombinantly produced in *E. coli* or yeast. The positively charged N-terminus of CPs is located inside the capsid and is responsible for attracting negatively charged RNA. CCMV VLPs undergo a reversible structural change in vitro as the buffer pH is altered. For instance, CCMV VLPs can be disassembled in a pH 7.5 buffer containing CaCl_2_, and after the viral genomic RNA is removed, the VLPs will reassemble at pH 5.0. This property is useful for packaging guest molecules [9]. CCMV VLPs can package a variety of heterologous ssRNAs in vitro, provided that their length falls within the range of 2,500 to 4,200 nt, which is similar to the CCMV genome size of around 3,000 nt [29].

**Fig. 3.**
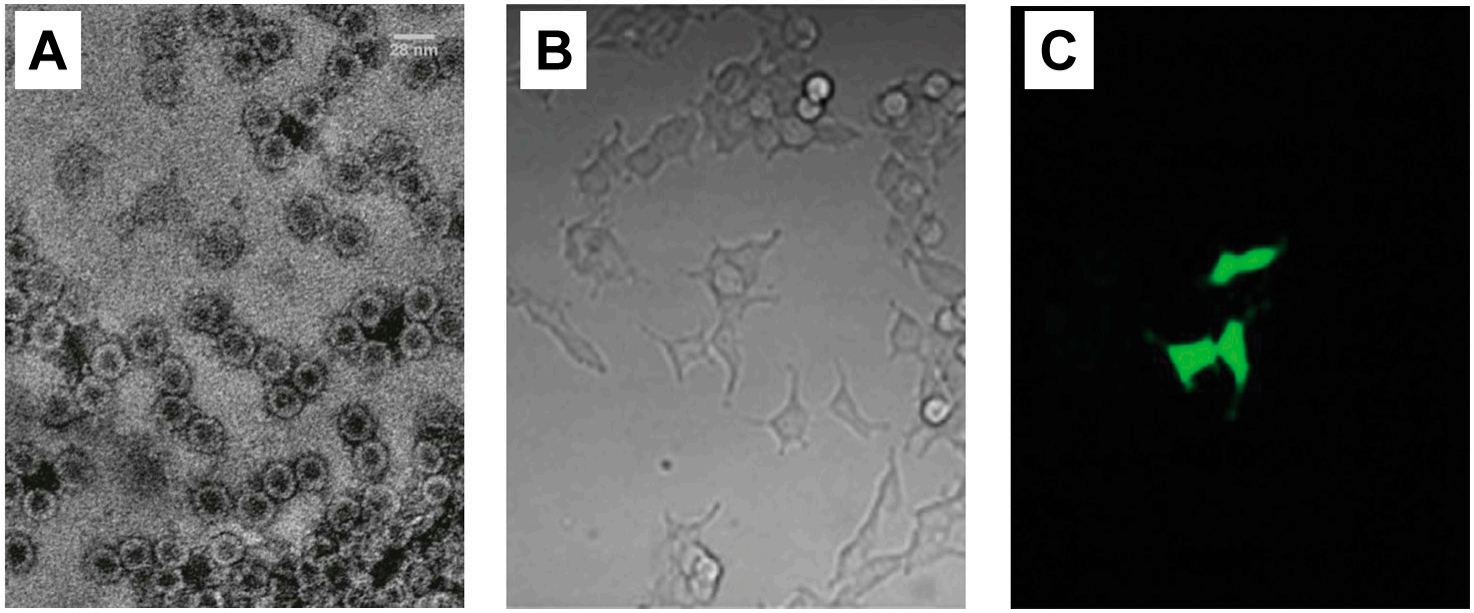
Intracellular mRNA delivery using CCMV VLPs. (A) Transmission electron microscopy image of EGFP mRNA-loaded CCMV VLPs. (B and C) Microscopy images (20× objective) of HEK293T cells incubated with EGFP mRNA-loaded CCMV VLPs. (B) The bright field. (C) The fluorescence field. Adapted with permission from reference [7].

Escareño et al. demonstrated the use of CCMV VLPs for the encapsulation and intracellular delivery of EGFP mRNA [7]. The recombinant CCMV VLPs could directly enter different eukaryotic cell lines without the aid of any transfection adjuvant. Specifically, CCMV CPs were prepared by disassembling CCMV that was obtained from infected cowpea plants. The in vitro transcribed EGFP mRNA was then coassembled with the CPs, leading to the formation of mRNA-loaded CCMV VLPs. After incubation of the VLPs with a few eukaryotic cell lines (HEK293T, Hela, and HK2), fluorescence of EGFP was observed in the cells, indicating successful translation of the delivered EGFP mRNA (Fig. 3). This study shows that CCMV VLPs are a good candidate carrier for mRNA delivery with the capability of protecting cargo mRNA and considerable translation efficiency. Due to the use of an in vitro package procedure, a packaging signal is not a prerequisite in this work, so a packing selectivity design was not included. In the case of mRNA packaging in living cells, a packaging signal would be required.

## Artificial VLPs

**Fig. 4.**
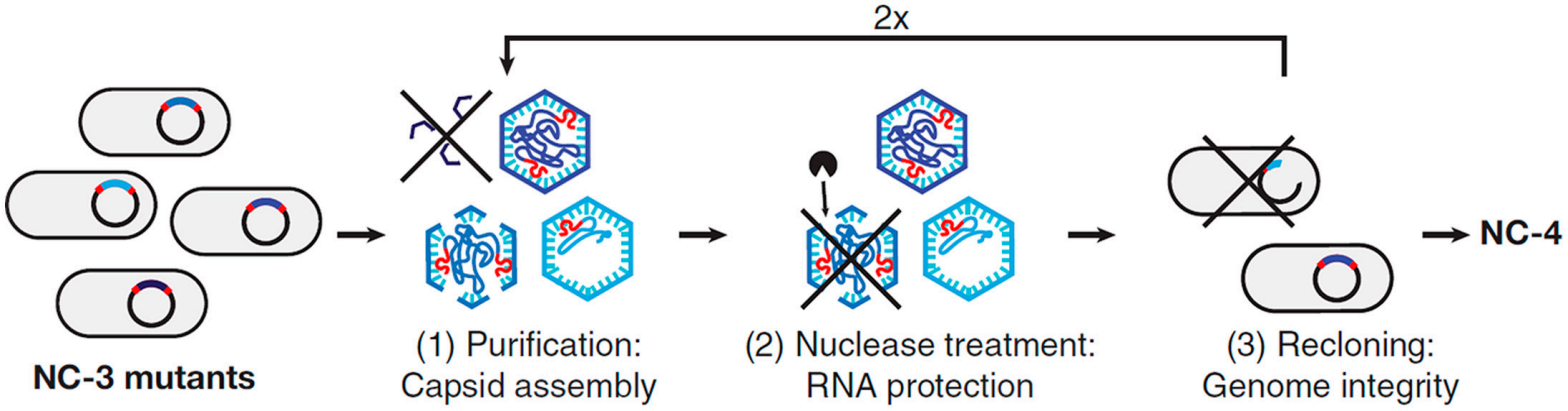
A directed evolution strategy to generate artificial VLPs capable of encapsulating and protecting their own encoding mRNA from nonviral PNCs. Adapted with permission from reference [31].

As an alternative to viral capsid, nonviral PNCs have also shown potential for mRNA encapsulation and delivery. For example, Hilvert et al. transformed a bacterial enzyme from AaLS, which lacks affinity for nucleic acids, into an artificial capsid by engineering and laboratory evolution. They appended cationic peptides to the proteins to enable specific recognition of packaging signals on cognate mRNAs. Then, the artificial capsid was optimized through directed evolution, resulting in expanded nucleocapsids that can encapsulate their own full-length mRNA in vivo and protect the cargo (size range: 400 to 2,000 nt) from enzymatic digestion [30]. Furthermore, they evolved the nucleocapsid (NC-3) by steadily increasing the selection stringency to improve the packaging properties. They succeeded in obtaining a larger 240-subunit icosahedral capsid (NC-4) that can package and protect multiple copies of its own mRNA with high encapsulation yields and specificity (Fig. 4) [31]. Notably, each such capsid can encapsulate 2.5 full-length mRNAs on average. This is a great improvement compared with its precursors (only 1 in 8 capsids can encapsulate the full-length RNA genome).

Besides naturally occurring nonviral proteins, de novo designed protein assemblies have also been reported to package mRNA. Baker et al. created artificial PNCs with positively charged inner surfaces based on their computationally designed 2-component protein assemblies. The PNCs can package their own encoding mRNA genomes. Using *E. coli* as the expression host, they obtained artificial VLPs with remarkably improved properties (i.e., genome packaging, RNA stability in blood, and in vivo circulation time) after several generations of directed evolution [8].

These studies demonstrate that directed evolution is a powerful tool for engineering natural and synthetic nonviral PNCs capable of packaging and protecting mRNA. However, there have been no reports on successfully using these artificial VLPs as carriers to deliver mRNA into mammalian cells and achieve expression. Equipping these artificial VLPs with abilities to cross intracellular barriers and release cargo mRNA is the major challenge in the following efforts.

## Challenges and Opportunities

Safe and effective delivery of mRNA remains one of the major challenges in realizing the promise of mRNA therapeutics. This review briefly summarizes the current progress in the utilization of PNCs for mRNA delivery in terms of PNC species, characteristics, mRNA encapsulation strategies, and delivery outcomes. These PNCs include VLPs, natural nonviral PNCs, and artificial PNCs. They share several advantages, e.g., convenient recombinant preparation, controllable disassembly and assembly, and readily surface modification. They also have some common shortcomings, e.g., relatively small volumes and low efficiency of mRNA translation. Besides, MS2 VLPs require harsh buffer conditions to disassemble and are vulnerable to genetic engineering, while CCMV VLPs disassemble at pH 7.5, which harms the stability of the VLPs under normal physiological conditions.

Although the idea of mRNA delivery using VLPs or PNCs was proposed nearly 2 decades ago, only a few PNCs have been explored currently, with limited reports on translation. Given the effective genome delivery by viruses in nature, especially that of nonenveloped RNA viruses, it is reasonable to expect a bright future of using PNCs for mRNA delivery, which deserves increased efforts to solve the existing problems. One of the greatest challenges is that PNCs are prone to be trapped in endosomes after cellular uptake, which causes failure or low efficiency in mRNA translation. In general, PNCs enter mammalian cells through endocytosis with the uptaken PNCs being intracellularly transported in endosomes and degraded in lysosomes. There are examples of endosome escape via functionalizing PNC surfaces with peptides rich in cationic or protonatable residues and examples of triggered cargo release via equipping PNCs with sensitivity to reducing conditions [17]. However, more efforts are needed to address this challenge. A second problem relates to the immunogenicity of PNCs. PNCs often cause strong immune responses when administrated in vivo. During such a process, exogenous PNCs are recognized as particulate antigens and taken up by different antigen-presenting cells, resulting in adaptive immune responses [32], which would reduce the efficacy of repeated dosing. Another big challenge is in vivo targeting. For applications such as mRNA-based protein replacement treatment and gene editing, PNCs are required to go to target cells, tissues, or organs. However, PNCs are often cleared by the reticuloendothelial system, resulting in short blood circulation time and inefficient accumulation in target sites. This is a common problem faced by nanomaterials [33]. Strategies established in nanotechnology to deal with this issue can be referred to [34]. Additionally, mRNA stability is also a concern when delivered by PNCs. Generally, it is expected that PNC encapsulation of mRNA offers protection from ribonuclease degradation. However, the study by Hilvert et al. [31] demonstrated that the holes in the PNC shell can allow ribonucleases of small molecular weights to get inside to digest the cargo mRNA. Further studies need to pay attention to this issue from perspectives of both the permeability of PNCs and the modification of mRNA.

Emerging technologies may offer new opportunities to overcome the obstacles to unlocking the potential of PNCs. Artificial intelligence (AI), which is revolutionizing all fields of science, would substantially improve the ability to learn from viruses, engineer existing PNCs, and design artificial PNCs with higher complexity and more predictable functions [35,36]. With the aid of AI, it is hopeful that one can design and construct novel PNCs that can (a) readily escape from the endosome after endocytosis or bypass endocytic pathways to enter cells and (b) disassemble in response to intracellular molecules to release mRNA cargoes into cytosol with much higher efficiencies. Also, to address the immunogenicity and in vivo targeting problems, it is now possible to design new PNCs with a self-protein as the building block to achieve carriers that can evade immune surveillance. Although AI provides powerful tools to direct the design and engineering of PNC nanostructures, the functions of the designed PNCs may still need testing and improvement in light of the complexity of biosystems. In this regard, directed evolution would be a complementary choice to AI. Despite not being an emerging technique, directed evolution, which follows Darwin's theory of evolution, has been a powerful tool in bioengineering. The breakthrough of PNC-based mRNA delivery technique would probably come from the combination of AI and directed evolution. Besides, techniques developed in synthetic biology [37] will hopefully enable the customizable evolution, biosynthesis, and modification of PNCs and mRNA of interest in recombinant systems and the spatiotemporal regulation thereof, which would promote the full biosynthesis of mRNA@PNC nanomedicines. In addition, concepts and findings in nanomedicine are equally applicable to the engineering of PNCs. For instance, strategies for designing nanomaterials with environmental responsiveness may help establish mechanisms to better control mRNA loading into and release from PNCs; the methods for surface engineering or camouflaging of nanoparticles may be useful for reducing the immunogenicity and in vivo targeting of PNCs. With the increasing convergence of different disciplines in the development of PNC-based materials, PNC carriers would open up a promising new avenue for mRNA therapeutic applications.
